# Oxaliplatin- or irinotecan-based chemotherapy for metastatic colorectal cancer in the elderly

**DOI:** 10.1038/sj.bjc.6601310

**Published:** 2003-10-14

**Authors:** T Aparicio, J Desramé, T Lecomte, E Mitry, J Belloc, I Etienney, S Montembault, L Vayre, C Locher, J Ezenfis, P Artru, M Mabro, S Dominguez, for AGEO

**Affiliations:** 1 Service d'Hépato-Gastroentérologie, Hôpital Bichat-Claude Bernard, 46 rue Henri Huchard, AP-HP, Paris 75018, France; 2 Clinique médicale, Hôpital d'instruction des armées du Val de Grâce, Paris 75005, France; 3 Service d'Hépato-Gastroentérologie, Hôpital Européen Georges Pompidou, AP-HP, Paris 75015, France; 4 Service d'Hépato-Gastroentérologie, Hôpital Ambroise Paré, AP-HP, Boulogne 92100, France; 5 Service d'Hépato-Gastroentérologie, Institut Gustave Roussy, Villejuif 94805, France; 6 Service d'Hépato-Gastroentérologie, Hôpital de Soissons, Soissons 02200, France; 7 Service d'Hépato-Gastroentérologie, Hôpital Cochin, AP-HP, Paris 75005, France; 8 Service d'Hépato-Gastroentérologie, Hôpital Henri Mondor, AP-HP, Créteil 94000, France; 9 Service d'Hépato-Gastroentérologie, Hôpital de Longjumeau, Longjumeau 91600, France; 10 Service d'Oncologie, Hôpital Saint Antoine, AP-HP, Paris 75011, France; 11 Service d'Oncologie, Hôpital Foch, Suresnes 92150, France; 12 Département Uro-Digestif, Centre Oscar Lambret, Lille 59020, France; 13 AGEO: ‘Association des gastroentérologues oncologues’ 26 Avenue des Gobelins, Paris 75013, France

**Keywords:** colon cancer, elderly, chemotherapy, oxaliplatin, irinotecan

## Abstract

The tolerance and efficacy of oxaliplatin and irinotecan for metastatic colorectal cancer are unknown in elderly patients. Methods. All consecutive patients over 74 years treated with oxaliplatin or irinotecan for metastatic colorectal cancer were enrolled. The tumour response was assessed every 2–3 months and toxicity was collected at each cycle according to World Health Organisation criteria. A total of 66 patients were enrolled from 12 centres. The median age was 78 years (range, 75–88 years); 39 patients had no severe comorbidity according to the Charlson score. In total, 44 and 22 patients received oxaliplatin or irinotecan, respectively, in combination with 5-fluororuracil±folinic acid or raltitrexed in 64 patients. A total of 545 chemotherapy cycles were administered in first (41%), second (51%) or third line (8%). A dose reduction occurred in 190 cycles (35%). Complete response, partial response and stabilisation occurred in 1.5, 20 and 47% of patients, respectively. The median time to progression and overall survival were 6.8 and 11.2 months in first line and 6.3 and 11.6 months in second line, respectively. Grade 3 and 4 toxicity occurred in 42% of patients: neutropenia 17%, diarrhoea 15%, neuropathy 11%, nausea and vomiting 8% and thrombopenia 6%. There was no treatment-related death. In selected elderly patients, chemotherapy with oxaliplatin or irinotecan is feasible with manageable toxicity.

Colorectal cancer is the second most frequent cancer in Europe (Bray *et al*, 2002) and 40% of patients are of age 74 years and more at diagnosis (Gatta *et al*, 1998). Nevertheless, patients over 75 years have been usually excluded from randomised clinical trials evaluating chemotherapy for advanced colorectal cancer (Trimble *et al*, 1994; Kohne *et al*, 2001). Few data are available on chemotherapy in elderly patients, but it has been suggested that 5-fluorouracil (5-FU)-based chemotherapy efficacy was comparable to that in younger patients (Stein *et al*, 1995; Chiara *et al*, 1998; Mabro *et al*., 1999; Popescu *et al*., 1999; Magne *et al*., 2002). Tolerance was comparable except in one study evaluating chemotherapy with bolus 5-FU (Stein *et al*, 1995). A survival benefit was demonstrated in one unpublished randomised study comparing 5-FU-based chemotherapy to best supportive care in elderly patients (Beretta *et al*, 1997). In the adjuvant setting, a pooled analysis of randomised trials with 5-FU-based chemotherapy confirmed the improvement of disease-free survival and overall survival (OS) with chemotherapy, even in patients over 70 years (Sargent *et al*, 2001).

Oxaliplatin (de Gramont *et al*, 2000) and irinotecan (Douillard *et al*, 2000), in combination with 5-FU–leucovorin, have demonstrated their efficacy in advanced colorectal cancer with a better tumour response rate than 5-FU and leucovorin alone. After 5-FU failure, irinotecan as second-line chemotherapy has demonstrated a survival advantage compared to best supportive care in advanced colorectal cancer (Cunningham *et al*, 1998). Combination therapy, however, has an increased toxicity compared to 5-FU alone, especially neutropenia, diarrhoea and neurologic toxicity with oxaliplatin and neutropenia, and diarrhoea with irinotecan.

No data from clinical trials are available about chemotherapy with oxaliplatin or irinotecan in patients aged 75 years and more with advanced colorectal cancer. We report the result of tolerance and efficacy of oxaliplatin- or irinotecan-based chemotherapy in a multicentre observational cohort of patients of age 75 years and more with an advanced colorectal cancer.

## PATIENTS AND METHODS

### Patients

Data were collected from 12 centres. Each investigator included all consecutives patients over 74 years treated with oxaliplatin or irinotecan for advanced colorectal carcinoma from January 1999 to June 2002. All patients had a histologically proven colorectal adenocarcinoma. All the regimens containing oxaliplatin or irinotecan were considered either in association with 5-FU/leucovorin, oral 5-FU or raltitrexed. Only the first regimen was considered for evaluation in patients receiving both oxaliplatin and irinotecan sequentially. Patients were divided into two groups: group 1 patients 75–79 years old and group 2 patients 80 years old and over. The following clinical and biological variables were recorded: age, sex, body mass index, primary localisation, sites of metastasis, World Health Organisation (WHO) performance status (PS), Charlson comorbidity score (Charlson *et al*, 1987), haemoglobin, creatinin clearance (according to Cockroft method), alkaline phosphatase and carcinoembryonic antigen.

### Chemotherapy

The chemotherapy regimens used were: FOLFOX 4 (leucovorin 200 mg m^−2^ day^−1^ as a 2-h infusion followed by bolus 5-FU 400 mg m^−2^ day^−1^ and a 22-h 5-FU infusion of 600 mg m^−2^ day^−1^, repeated for 2 consecutive days every 2 weeks. Oxaliplatin 85 mg m^−2^ was given on day 1 as a 2-h infusion concurrent with leucovorin) (de Gramont *et al*, 2000), FOLFOX 6 (oxaliplatin 100 mg m^−2^ was infused with leucovorin 400 mg m^−2^ as a 2-h infusion on day 1, followed by bolus 400 mg m^−2^ and a 46-h infusion 2400 mg m^−2^ of 5-FU, every 2 weeks) (Maindrault-Goebel *et al*, 2000), FOLFOX 7 consisted of the same regimen with oxaliplatin 130 mg m^−2^ (Maindrault-Goebel *et al*, 2001), ELOXFU-3 (leucovorin 200 mg m^−2^ as a 2-h infusion followed by bolus 5FU 400 mg m^−2^ and a 48-h infusion of 2400 mg m^−2^ and oxaliplatin 130 mg m^−2^ on day 1 every 3 weeks) (Ducreux *et al*, 1999), LV5FU2-irinotecan (leucovorin 200 mg m^−2^ day^−1^ as a 2-h infusion followed by bolus 5-FU 400 mg m^−2^ day^−1^ and a 22-h 5-FU infusion of 600 mg m^−2^ day^−1^, repeated for 2 consecutive days every 2 weeks. Irinotecan 180 mg m^−2^ was given on day 1 as a 1.5-h infusion concurrent with leucovorin) (Douillard *et al*, 2000).

According to evidence-based medicine, treatment was continued until disease progression or unacceptable toxicity occurred or until a patient chose to discontinue it.

### Study parameters

When measurable, tumour response was assessed with computed tomography (CT) examination every 2–3 months according to WHO criteria. A complete response was defined as the complete disappearance of all clinically assessable disease, and a partial response was defined as a decrease of at least 50% of the sum of the products of the diameters of measurable lesions. Stable disease was defined as a decrease of less than 50% or an increase of less than 25% of measurable lesions, and progressive disease was defined as an increase of at least 25% of measurable lesions or the appearance of new malignant lesion(s). Progression-free survival (PFS) was defined as the time interval from the beginning of oxaliplatin- or irinotecan-based chemotherapy to the date of disease progression or, if the patient died without evidence of progression, to the date of death. OS lasted from the date of the beginning of oxaliplatin- or irinotecan-based chemotherapy to the date of death or the date when the patient was seen for the last time. Toxicities were collected at each cure according to WHO criteria.

### Statistical analysis

Since this was not a randomised study, only patient characteristics at inclusion were statistically compared, using *χ*^2^ test for categorical variables and Student's *t*-test for continuous variables. OS and PFS were calculated using the Kaplan–Meier method. For all statistical tests, *P*-values less than or equal to 0.05 were considered significant.

## RESULTS

### Patients and treatment

Data from 66 consecutive patients treated from January 1999 to June 2002 were collected. Patient characteristics are presented in Table 1

**Table 1:** Patient characteristics

**Patients characteristics**	**All patients, *n*=66 (%)**	**Group 1, *n*=48 (%)**	**Group 2, *n*=18 (%)**	**First line, *n*=27 (%)**	**Second line, *n*=34 (%)**
*Sex*					
Male	39 (59)	25 (52)	14 (78)*	15 (56)	20 (59)
Female	27 (41)	23 (48)	4 (22)	12 (44)	14 (41)
*Site of primary tumour*					
Colon	52 (79)	38 (79)	14 (78)	20 (74)	27 (79)
Rectum	14 (21)	10 (21)	4 (22)	7 (26)	7 (21)
*No. of metastatic site(s)*					
1	38 (58)	30 (62)	8 (44)	16 (59)	19 (56)
2	19 (29)	11 (23)	8 (44)	8 (30)	10 (29)
>2	9 (14)	7 (15)	2 (12)	3 (11)	5 (15)
*Charlson comorbidity index*					
0	39 (59)	29 (60)	10 (55)	16 (59)	21 (62)
1–2	18 (27)	13 (27)	5 (28)	6 (22)	10 (29)
>2	9 (14)	6 (13)	3 (17)	5 (19)	3 (9)
*Performance status*					
0	13 (20)	11 (23)	2 (11)	3 (11)	8 (24)
1	31 (47)	22 (46)	9 (50)	14 (52)	14 (41)
2	19 (29)	15 (31)	4 (22)	8 (30)	11 (32)
3	3 (4)	0 (0)	3 (17)**	2 (7)	1 (3)
Mean creatinin clearance (ml min^−1^)	58.9±1.8	61.4±2.1	52±3.5***	62±2.5	57±2.6
Mean haemoglobin (g dl^−1^)	12±0.2	11.8±0.2	12.5±0.3	11.8±0.2	12.1±0.3
CEA <5 (ng ml^−1^)	11 (17)	9 (19)	2 (11)	5 (18)	6 (18)
>5–50 ng ml^−1^	19 (29)	13 (27)	6 (33)	7 (26)	9 (26)
>50 ng ml^−1^	31 (47)	22 (46)	9 (50)	15 (56)	15 (44)
Not done	5 (7)	4 (8)	1 (6)	0	4 (12)
*Alkaline phosphatase*					
<*N*	42 (64)	34 (71)	8 (45)	19 (70)	20 (59)
>*N*–2*N*	11 (17)	5 (10)	6 (33)	4 (15)	5 (15)
>2*N*	10 (15)	6 (13)	4 (22)	4 (15)	6 (17)
Not done	3 (4)	3 (6)	0****	0	3 (9)

* *P*=0.058.

** *P*=0.025.

*** *P*=0.04.

**** *P*=0.044.

. The median age was 78 years (range, 75–88 years). In all, 48 patients were 75–79 years old (group 1) and 18 were aged 80 or over (group 2). For groups 1 and 2, the median ages were, respectively, 77 years (range, 74–79 years) and 81 years (range, 80–88 years). Of the 66 patients, 39 had no severe comorbidity according to the Charlson index. No significant difference for sex, site of primary tumour, number of metastasic sites and comorbidity were observed between patients of groups 1 and 2. Group 2 patients had a significantly poorer PS. Creatinin clearance was significantly lower in group 2 than in group 1 (Table 1). There was no significant difference in baseline characteristics between patients treated in first line and patients treated in second line (Table 1).

Out of the 66 patients, 44 received an oxaliplatin-based regimen and 22 received an irinotecan-based regimen. A total of 60 patients were treated with a combination of oxaliplatin or irinotecan with 5-FU/leucovorin (Table 2

**Table 2:** Chemotherapy characteristics

**Chemotherapy regimens**	**All patients, *n*=66 (%)**	**Mean number of cycles^a^**	**Group 1, *n*=48 (%)**	**Mean number of cycle**	**Group 2, *n*=18 (%)**	**Mean number of cycle**
LV5FU2-irinotecan	21 (32)	10.9±1.3	15 (31)	10.7±1.4	6 (33)	11.5±3.2
FOLFOX 4	20 (30)	8.2±0.7	17 (36)	8.8±0.8	3 (17)	5.3±1.4
FOLFOX 6	12 (18)	7.8±1	8 (17)	8±1.4	4 (22)	7.5±1.8
ELOXFU 3	5 (8)	3.4±0.8	4 (8)	3.5±1	1 (6)	3.0
FOLFOX 7	2 (3)	7.0±1	2 (4)	7.0±1	—	—
Oxaliplatin monotherapy	2 (3)	5.0±2	2 (4)	5.0±2	—	—
Others^b^	4 (6)	4.0±1	—	—	4 (22)	4.0±1
Overall		10.8±0.8		10.2±0.8		12.5±2.2

a LV5FU2-irinotecan, FOLFOX 4, FOLFOX 6 and FOLFOX 7 are biweekly regimens; ELOXFU 3, oxaliplatin monotherapy and other regimens are three-weekly regimens.

b One patient was treated with an association of raltitrexed and oxaliplatin, two patients with an association of capecitabine and oxaliplatin and one patient with an association of raltitrexed and irinotecan.

). Patients received oxaliplatin- or irinotecan-based regimen mainly as second-line treatment (51%) after failure of 5-FU (Table 3

**Table 3:** Repartition of oxaliplatin or irinotecan chemotherapy line

**Chemotherapy line**	**All patients, *n*=66 (%)**	**Group 1, *n*=48 (%)**	**Group 2, *n*=18 (%)**
First line	27 (41)	20 (42)	7 (39)
Oxaliplatin	19	14	5
Irinotecan	8	6	2
			
Second line	34 (51)	26 (54)	8 (44)
Oxaliplatin	21	17	4
Irinotecan	13	9	4
			
Third line	5 (8)	2 (4)	3 (17)
Oxaliplatin	4	2	2
Irinotecan	1	—	1

).

Among the 545 cycles of chemotherapy administered, 190 cycles (35%) were carried out with a dose reduction of 126 (31%) in group 1 and 64 (48%) in group 2.

### Toxicity

In all, 28 (42%) patients experienced grade 3 and 4 toxicity (Table 4

**Table 4:** Maximum toxicity per patient

**Grade 3 and 4 toxicity**^a^	**All patients, *n*=66 (%)**	**Oxaliplatin-based regimen, *n*=44 (%)**	**Irinotecan-based regimen, *n*=22 (%)**	**Group 1, *n*=48 (%)**	**Group 2, *n*=18 (%)**
Any grades 3 and 4 (except alopecia)	28 (42)	16 (36)	12 (54)	20 (42)	8 (44)
Neutropenia	11 (17)	6 (14)	5 (23)	8 (17)	3 (17)
Thrombopenia	4 (6)	4 (9)	0	2 (4)	2 (11)
Anaemia	1 (1.5)	1 (2)	0	1 (2)	0
Nausea and vomiting	5 (8)	3 (7)	2 (9)	4 (8)	1 (6)
Diarrhoea	10 (15)	4 (9)	6 (27)	7 (15)	2 (11)
Stomatitis	2 (3)	2 (5)	0	2 (4)	0
Neurologic	7 (11)	7 (16)	0	5 (10)	2 (11)
Treatment interruption for toxicity	11 (17)	8 (18)	3 (14)	9 (19)	2 (11)
Number of delayed cycles for toxicity/total number of cycles	38/545 (7)	24/309 (8)	14/236 (6)	27/411 (7)	11/134 (8)

a According to WHO grade.

). The main severe toxicities were neutropenia in 11 patients and diarrhoea in 10 patients. The toxicity pattern was similar in groups 1 and 2. Definitive treatment interruption for toxicity occurred in four of the nine (44%) patients with a Charlson score higher than 2 and in seven of the 57 (12%) patients with a Charlson score equal or less than 2.

### Objective tumoral responses

Three patients (4.5%) were not evaluable for tumoral response. Objective responses were observed in 14 patients (21%) (Table 5

**Table 5:** Tumoral responses

	**All patients, *n*=66 (%)**	**First line, *n*=27 (%)**	**Second line, *n*=34 (%)**	**Group 1, *n*=48 (%)**	**Group 2, *n*=18 (%)**
Complete response	1 (1.5)	1 (4)	0	0	1 (6)
Partial response	13 (20)	9 (33)	4 (12)	11 (23)	2 (11)
Stable disease	31 (47)	12 (44)	17 (50)	20 (42)	11 (61)
Progression	18 (27)	4 (15)	12 (35)	14 (29)	4 (22)
Not evaluable	3 (4.5)	1 (4)	1 (3)	3 (6)	0

). Tumour control defined as an objective response or stabilisation was obtained in 44 patients (67%).

### PFS and OS

**Figure 1:**
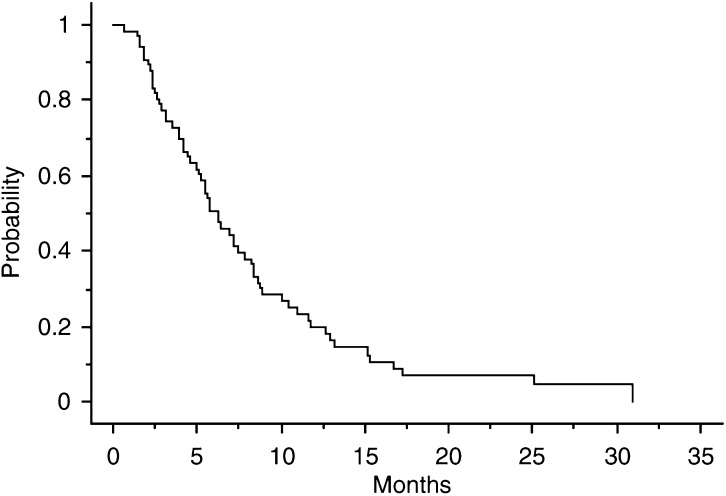
PFS curve.

**Figure 2:**
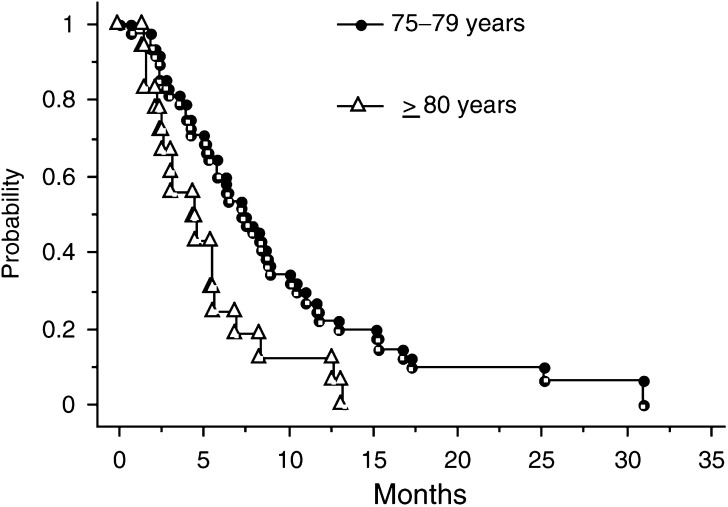
PFS curves according to age group.

**Figure 3:**
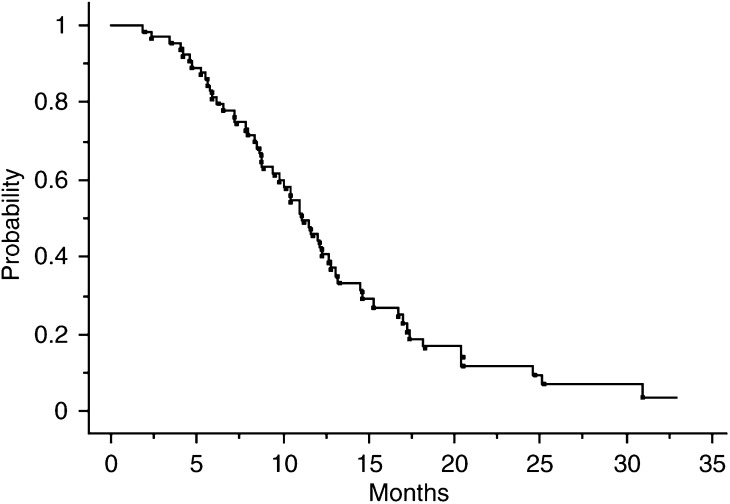
OS curve.

**Figure 4:**
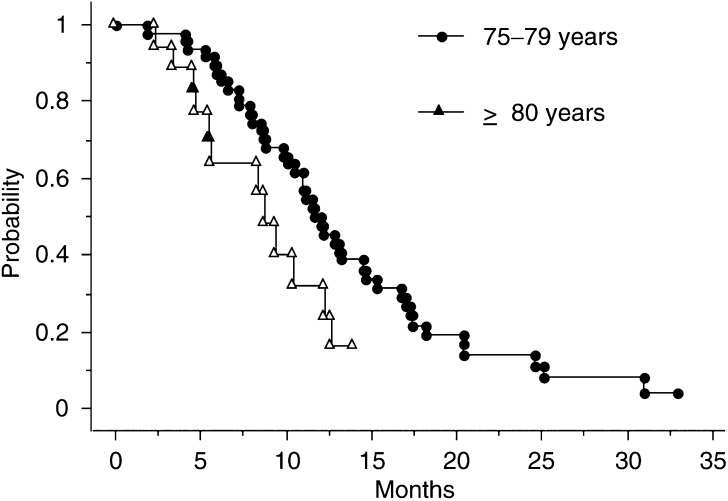
OS curves according to age group.

For all patients, the median PFS was 6.3 (range, 1–30.9) months (Figure 1

). PFS was 4.5 (range, 1.4–13.2) months for group 2 patients and 7.3 (range, 1–30.9) months for group 1 patients (Figure 2

). The median OS was 11.8 (range, 1.8–32.8) months (Figure 3

). The median OS was 9.9 (range, 2.3–13.9) months for group 2 patients and 12.1 (range, 1.8–32.8) months for group 1 patients (Figure 4

). The median OS from the beginning of the first-line chemotherapy was 16.7 (range, 4–42.3) months. The survival rate from the beginning of the first-line chemotherapy was 72% at 1 year and 34% at 2 years.

For the 27 patients treated in first line, the median PFS was 6.8 months (range, 1–29.2) and the median OS was 11.2 months (range, 4–29.2). For the 34 patients treated in second line, the median PFS was 6.3 months (range, 1–30.9) and the median OS was 11.6 months (range, 1.8–32.8). The median OS from the beginning of the first-line chemotherapy was 20.6 months (range, 5.8–42.3) in these 34 patients.

### PS and weight improvement

Among the 53 patients with an initial PS >1, six were not evaluable for PS evolution and 15 (32%) had a PS improvement. A total of 28% of all the patients had a gain in weight during the treatment.

## DISCUSSION

This is the first study of tolerance and efficacy of oxaliplatin- and irinotecan-based chemotherapy in elderly patients. Our results show that oxaliplatin- or irinotecan-based chemotherapy in elderly patients is feasible and safe.

A total of 42% of the patients experienced severe toxicity without treatment-related death. Previous retrospective studies evaluating 5-FU+leucovorin regimens in an elderly population reported severe toxicity in 20% of patients treated with the LV5FU2 regimen (Mabro *et al*, 1999) and in 58% of patients treated with the 5-FU bolus regimen (Stein *et al*, 1995). We report severe neutropenia in 14 and 23% of patients treated with oxaliplatin and irinotecan, respectively. Previous studies in nonelderly patients reported severe neutropenia in 36–41% (Andre *et al*, 1999; de Gramont *et al*, 2000) with oxaliplatin and 29–54% (Douillard *et al*, 2000; Saltz *et al*, 2000) with irinotecan. In our study, severe neuropathy occurred in 16% of patients treated with oxaliplatin; previous studies reported severed neuropathy in 18 and 20% (Andre *et al*, 1999; de Gramont *et al*, 2000). Severe diarrhoea occurred in 27% of patients treated with irinotecan. Previous studies reported severe diarrhoea in 22–44% (Douillard *et al*, 2000; Saltz *et al*, 2000). A full dose of chemotherapy was administered in 65% of the cycles. Previous studies in nonelderly patients reported a dose intensity of 73% for oxaliplatin (de Gramont *et al*, 2000) and 72–93% for irinotecan (Douillard *et al*, 2000; Saltz *et al*, 2000) in first-line chemotherapy. The frequent dose reduction observed in our report, especially in group 2, could partly explain the mild toxicity.

The overall objective response rate was 22%; 38% in first-line treatment. The response rates reported in previous studies in elderly patients treated with a 5-FU- or raltitrexed-based regimen were 18% (Chiara *et al*, 1998), 22% (Feliu *et al*, 2002), 24% (Popescu *et al*, 1999), 26% (Magne *et al*, 2002) and 44% (Mabro *et al*, 1999). The response rate in nonelderly patients treated in first line with an oxaliplatin- or irinotecan-based regimen is about 50% (de Gramont *et al*, 2000; Douillard *et al*, 2000; Saltz *et al*, 2000). In second line, the response rates ranged from 17% (Rougier *et al*, 1997) to 46% (de Gramont *et al*, 1997). The response rates observed in our study in first-line treatment seem to be higher than those generally obtained with 5-FU alone in elderly patients and comparable to those obtained with oxaliplatin or irinotecan in nonelderly patients. In second line, the response rates seem to be lower than those published with oxaliplatin or irinotecan in nonelderly patients.

We observed a median OS of 11.2 months in first line. It was 9.6 (Feliu *et al*, 2002), 9.7 (Popescu *et al*, 1999), 16.4 (Mabro *et al*, 1999) and 14 months (Magne *et al*, 2002) in previous studies where elderly patients were treated with a 5-FU- or raltitrexed-based regimen. Nevertheless, although we did not observe a better OS with oxaliplatin or irinotecan than in previous studies with 5-FU or raltitrexed, it must be pointed out that in all these studies the median age was younger and only few patients over 79 years were considered. We have suggested that age over 79 years is associated with poor survival. This population represents 27% of the patients in our study and could explain the poor OS observed. Nevertheless, the survival observed in our elderly patients is poorer than the one reported in prospective studies in younger patients in first-line treatment (de Gramont *et al*, 2000; Douillard *et al*, 2000; Saltz *et al*, 2000). In patients treated in second line with oxaliplatin or irinotecan, we observed an 11.6 month median survival from the beginning of second line and a 20.6 month median survival from the beginning of first line. These data prompted us to use a second-line chemotherapy in the elderly after first-line failure. Nevertheless, the discrepancy between OS of patients treated in first line with oxaliplatin or irinotecan and OS from the beginning of chemotherapy in patients treated in second line with oxaliplatin or irinotecan suggest that tumours of patients treated in second line had a slower growth rate.

The median PFS in first line was 6.8 months, which is comparable with PFS reported in younger patients: 9 months with oxaliplatin (de Gramont *et al*, 2000) and 6.7–7 months with irinotecan (Douillard *et al*, 2000; Saltz *et al*, 2000). An improvement of PS was noted in 32% of the patients with initial degraded PS; this is comparable with the 42% of improvement of PS reported with oxaliplatin in younger patients (de Gramont *et al*, 2000). In elderly patients treated with 5-FU alone, PS improvement varied from 26 to 56% (Mabro *et al*, 1999; Magne *et al*, 2002). Nevertheless, it is necessary to evaluate the influence of chemotherapy on quality of life and patient autonomy in a prospective study on elderly patients. In our study, the majority of the patients had no or little severe comorbidity. This suggests that the population is selected. This is the first study using the Charlson comorbidity score for elderly patients treated for metastatic colorectal carcinoma. A prognostic value of Charlson >2 was suggested for the occurrence of definitive treatment interruption for toxicity. A stratification according to the Charlson comorbidity score should be considered in prospective studies in elderly patients.

We conclude that chemotherapy with oxaliplatin or irinotecan in selected elderly patients is feasible with manageable toxicity. Improvements of PS and prolonged PFS and OS were obtained, but the benefit is weaker after 79 years. Moreover, second-line chemotherapy should be considered whenever it is possible in elderly patients. Prospective studies are needed to establish the benefit of the different chemotherapy regimens for quality of life and autonomy in elderly patients.
